# An Atypical Case of Rickettsial Spotted Fever Myocarditis Mimicking Weil's Disease

**DOI:** 10.1155/2019/9620245

**Published:** 2019-07-08

**Authors:** N. D. B. Ehelepola, G. D. N. R. Kumara, S. A. C. S. Sapurugala, W. M. N. P. Buddhadasa, Wasantha P. Dissanayake

**Affiliations:** Teaching (General) Hospital–Kandy, Kandy, Sri Lanka

## Abstract

A 65-year-old previously healthy male presented to us on the fourth day of a febrile illness with headache, arthralgia, myalgia, nausea, cough, chest pain, sore throat, and passing of watery stools and dark urine with a history of exposure to leptospirosis during a dengue outbreak. On examination, there was dehydration and hypovolemia, and an ultrasound scan revealed capillary leakage. His liver transaminases, serum creatine, blood urea, C-reactive protein, and neutrophil percentage were high, and thrombocytopenia was present. Moreover, myocarditis has been detected too. Supportive therapy with intravenous ceftriaxone was administered, considering possible Weil's disease or dengue hemorrhagic fever with secondary bacterial infection. Serological tests, performed later, diagnosed him with a *Rickettsia conorii* infection and excluded dengue, leptospirosis, and hantavirus infections. Repeat 2D echocardiograms showed mild improvement of his cardiac failure after one month and a more improvement after eight months. Clinical features of the rickettsial spotted fever group (SFG) and leptospirosis overlap. Leptospirosis is common; thus, the risk of overlooking SFG and diagnosing leptospirosis is likely. Tests for differentiation are unavailable in Sri Lankan hospitals and in many other developing countries. Empirical doxycycline in suspected cases of SFG infections in areas where rickettsioses are prevalent can save lives as in this case.

## 1. Introduction

Leptospirosis and rickettsial spotted fever group (SFG) are emerging and reemerging bacterial zoonotic infections in Sri Lanka and globally [1–5]. SFG is a group of vector-borne diseases transmitted by ticks and mites which are ectoparasites of mammals, while leptospirosis is directly transmitted by rodents and other mammals. In both diseases, people are incidental hosts [1, 5]. Both SFG and leptospirosis have protean clinical manifestations, and their clinical features can overlap which makes the clinical differentiation between them impossible sometimes [5, 6]. The vast majority of hospitals in low- and middle-income countries do not have facilities for serological or molecular genetic diagnosis of both these diseases; thus, clinicians in poorly resourced settings are compelled to depend on clinical features and basic investigations for differentiation [5, 7]. Leptospirosis is more prevalent in Sri Lanka as well as in most other countries; hence, most healthcare workers are familiar with it [5, 7, 8]. Rickettsioses are sparser and have no pathognomonic signs [6]. Therefore, there is always a risk of overlooking SFG and diagnosing leptospirosis in atypical SFG infections, especially in developing countries where the burden of these diseases is high [7]. Severe forms of leptospirosis with multiorgan failure (Weil's disease) have a high case fatality rate, and the same is true for severe SFG [1, 5]. Both diseases are treatable; hence, early correct diagnosis can reduce mortality and morbidity. Moreover, correct diagnosis is important for preventive work. Presence of rickettsial SFG in Sri Lanka especially in the central hill country is being increasingly reported after 2001 [2]. Nevertheless, the responsible rickettsial species have not been clearly identified yet [2]. Brown dog tick (*Rhipicephalus sanguineus*), the usual vector of SFG rickettsioses in Asia including Sri Lanka, is the second most abundant tick species in domestic animals and the most common species infecting wild animals, present in 15 species of wild animals in Sri Lanka as shown in one study [9]. A study of 25 tick species, infesting people and animals of Sri Lanka, has identified four tick species, namely, *Rhipicephalus sanguineus* from a dog, *Amblyomma testudinarium* from a wild boar, *Amblyomma clypeolatum* from a star tortoise, and *Amblyomma javanense* from a pangolin, having rickettsial infections. The first three tick species infest people as well [10].

Here, we present an atypical case of SFG with myocarditis, which we initially managed as leptospirosis and later correctly diagnosed to be a case of SFG and successfully managed, and lessons to be learned from this case.

## 2. Case Presentation

A 65-year-old Sinhalese man from Kandy district in the central hill region of Sri Lanka presented to us on day 4 of the illness with high fever, headache, arthralgia, myalgia, nausea, dry cough, chest pain, sore throat, and passing of watery stools and dark urine. Upon examination, he was dehydrated, temperature was 38°C, pulse rate was 120/minute, and blood pressure was 70/40 mm·Hg, and there were few basal crepitations on his right lung with mild epigastric tenderness. There was no lymphadenopathy or any other remarkable findings. He was admitted during the height of an outbreak of dengue, so suspecting dengue hemorrhagic fever (DHF), inward hematocrit was checked and it was 48%. A bedside ultrasound scan of the abdomen demonstrated edema of the gall bladder wall and a small amount of free fluid. He was resuscitated with intravenous fluid, and blood was sent for workup suspecting DHF or leptospirosis (he owns a small dairy farm and a pet dog, and leptospirosis is common locally). As illustrated in Table 1, his liver transaminases, serum creatine, and blood urea levels were high, and the percentage of neutrophils remained high in the full (complete) blood count.

His CRP levels remained high from day 4 to day 6. Therefore, our first differential diagnosis was leptospirosis, and the second was DHF with possible secondary bacterial infection [11]. We maintained fluid balance and supportive therapy as for DHF according to the current national guidelines which are broadly similar to the World Health Organization (WHO) 2009 guidelines [12] and added intravenous (IV) ceftriaxone 1 g twice daily until day 11 to cover leptospirosis. The common secondary bacterial infection in DHF patients we see here is IV cannula site infections or skin infections with *Staphylococcus aureus*. There was no evidence of such infections in this patient, and the neutrophil percentage was above 90% even in the results of the first test done on admission. His first ECG (EKG) showed inverted *T* wave in leads 5 and 6 and in the repeated ECGs that was present only in lead 5. His troponin I levels were also high. A 2D echocardiogram revealed global hypokinesia with a grade II aortic regurgitation. Nevertheless, we were unable to exclude any preexisting mild aortic regurgitation as this patient had never undergone an echocardiogram before. The patient was reassessed by a consultant cardiologist on day 8, the same findings plus a grade III mitral regurgitation and a grade II tricuspid regurgitation were detected, and the left ventricular ejection fraction (LVEF) was 30%. Congestive cardiac failure due to possible leptospiral myocarditis was diagnosed. Dobutamine and furosemide infusions were administered between days 4 and 10, and supportive therapy continued. Considering his lung and cardiac involvement, 1 g of intravenous methylprednisolone was administered daily for 3 days starting from day 5 and followed by 40 mg oral prednisolone daily. His blood sugar level rose after that and was controlled by subcutaneous soluble insulin injections. Few months before his admission, we received several influenza A H1N1 patients. Influenza A H1N1 also can cause similar symptoms. Until the report of throat swab for influenza A and B viral RNA becomes available, the patient was given oral oseltamivir 75 mg twice daily from days 4–9. Throat swabs were negative for influenza A and B (report arrived later). Test for both IgM and IgG against the dengue virus was negative on day 6. This patient was never icteric. There was no conjunctival suffusion and myalgia in his calf and was minimal in the back of the neck. Considering absence of those common clinical features of Weil's disease, we have tested for possible rickettsial infection despite the absence of any inoculation eschar (tache noire) or any other cutaneous lesions. We started 100 mg doxycyline twice on day 6 and continued 100 mg daily for a subsequent 6 days. On day 8, in the immunofluorescence antibody test (IFAT) in the blood sample with 1 : 32 dilution, IgG against *Rickettsia conorii* was positive (++), and with 1 : 48, 1 : 96, and 1 : 192 dilutions, IgM against *Rickettsia conorii* was positive. However, tests for further dilutions were not performed due to shortage of test kits. On day 13, the ELISA test for IgM against leptospirosis in 1 : 100 dilution became negative. The patient was discharged on day 14 on 40 mg oral furosemide and 25 mg spironolactone twice a day and reassessed after a month. He had only a mild dyspnea and moderate tiredness while performing his daily routine, and a repeated 2D echocardiogram showed a global left ventricular hypokinesia, but the right ventricular functions were normal. A grade I mitral regurgitation and mild aortic regurgitation were detected, and the LVEF was 35%. After that, he defaulted follow-up but continued taking same drugs irregularly. A 2D echocardiogram, repeated after eight months, showed apical, apicoseptal, and anterior hypokinesia and reduced muscle mass of the left ventricle without scarring and LVEF of 40%. A grade I aortic regurgitation was present. However, he has been continuing his daily routine without any symptoms.

## 3. Discussion

The diagnosis of the patient was tricky because as explained in Introduction, according to his symptoms, signs, and results of initial basic blood investigations, both DHF with secondary bacterial infection and leptospirosis (that are common in this country and in other tropical countries) had been likely [1, 5–7]. These clinical features were compatible with hantavirus, influenza A H1N1 virus infections, and rickettsial infections as well although they are less common [6, 7]. We failed to perform IgG and IgM titers and looked for a fourfold rise because of shortage of test kits. A fourfold rise of titers in paired samples is widely accepted as a confirmatory test for the diagnosis of rickettsioses. Nevertheless, according to the test kit manufacturer's literature (Vircell microbiologists, Granada, Spain), a positive result for IgM in 1 : 192 dilution is good enough for presumptive diagnosis of recent *Rickettsia conorii* infection, and it has to be confirmed by other supportive test results and the clinical picture. Considering the whole picture of this case, we believe our patient was very likely to have a *Rickettsia conorii* infection, but we could not confirm that diagnosis with standard serological tests. The majority of Sri Lankans obtain treatment for severe infections from state hospitals like ours that provide services free of charge, and to the best of our knowledge, none of the state hospitals in the country (and private hospitals) have in-house facilities to perform all the serological tests we performed to arrive at correct diagnosis. The situation to the best of our knowledge has been the same in many other developing nations where these infections are prevalent. Clinicians, handicapped by lack of tests for differentiation, are prone to arrive at incorrect diagnoses (i.e., overlooking rickettsial SFG for leptospirosis and dengue), and preventive care workers cannot take any action to prevent the spread of the rickettsial SFG as they are unaware of the emerging rickettsial infections without information from clinicians. In one study performed at another Sri Lankan teaching hospital, 10 out of 31 patients managed as Chikungunya and followed up were later serologically proven to be misdiagnosed cases of acute rickettsioses [13]. Cases of rickettsial SFG are rarely diagnosed in Sri Lanka. Nonetheless, one serological survey showed out of 883 febrile patients who had not been suspected of rickettsial infections, 17.7% had acute rickettsioses and 38.7% had exposure to spotted fever and/or typhus group rickettsioses [14]. One seroprevalence study regarding rickettsioses performed in Sri Lanka showed 37.3–85.2% of tested asymptomatic people in different regions around the country were seropositive, and IgG against *R. conorii* was the most prevalent [4]. Brown dog tick is the usual vector of SFG rickettsioses, and 21%–55% of domestic dogs tested from three localities close to Kandy were positive for IgG against *R. conorii* [15]. These indicated that rickettsial SFG infections are common here but are underdiagnosed (usually get misdiagnosed as other infections). A high index of suspicion directed us towards the correct diagnosis. Considering this patient's clinical features, most colleagues we know would have managed him for Weil's disease or DHF with a secondary bacterial infection. Some of us would have managed his daily fluid balance as for DHF and added an antibiotic like cloxacillin that covers *Staphylococcus aureus*, the most likely bacteria known to cause secondary infections in dengue [11]. Some of us whose first differential diagnosis was Weil's disease would have treated him with ceftriaxone or benzylpenicillin (penicillin G). Because of the patient's lack of any inoculation eschar or any other cutaneous lesions and as he is from a locality where rickettsia infections are not usually reported and without any history of a tick bite, most doctors we know would not have suspected rickettsia infection seriously or added doxycycline. Notwithstanding the fact that most local doctors give great emphasis to inoculation eschar in suspected cases of rickettsioses, and in one study conducted at a nearby hospital, none of the 105 serologically confirmed rickettsioses patients had had an eschar [3]. Inoculation eschars were also rare in *R. conorii* subsp. *indica* infections reported from neighboring India, but that usually resulted in a purpuric rash [6]. *Rickettsia helvetica* is known to cause noneruptive infections like in our case [6]. Few cases of myocarditis, pericarditis, atrial fibrillation, and ectasia of coronary arteries have been reported due to *R. conorii* infections worldwide [16, 17]. According to Colomba et al. [16], there had been only one case report of myocarditis due to SFG rickettsia infection from Asia up to 2016. Nevertheless, we found a past report in a local journal on myocarditis due to rickettsioses from the Western Province of Sri Lanka, but the infecting organism (whether *Orientia tsutsugamushi* or SFG rickettsia) had not been clearly mentioned there [18]. There is a very recent case report from Kandy, Sri Lanka, on myocarditis due to *R. conorii* infection [19]. He was successfully treated with intravenous chloramphenicol, hydrocortisone, and supportive therapy [19]. These cases reiterate that myocarditis is a rare but serious complication of rickettsial SFG, and it is treatable. After adding doxycycline on day 6, his symptoms and CRP levels improved swiftly. It further confirms the diagnosis of rickettsioses. A rational use of antibiotics and minimal use of a number of antibiotics to prevent bacterial resistance are a very important current topic. This issue is more acute in low- and middle-income nations. Nonetheless, to prescribe the most appropriate antibiotic in patients as in this case and to manage patients without endangering their lives, the clinicians need to have access to reliable confirmatory laboratory tests promptly and that are also scarce in the developing nations like Sri Lanka. We believe until rapid in-house serological tests become available widely, there is a place for empirical doxycycline in hospitalized febrile patients, when there is suspicion of rickettsioses as a differential diagnosis, in areas rickettsioses is present such as in the central hill country of Sri Lanka [20]. In patients diagnosed as Weil's disease based on clinical features and the results of basic investigations from the central hill country of Sri Lanka, it may be worthy of including oral doxycycline to IV ceftriaxone or penicillin G to cover possible rickettsioses if there are reasons to suspect that as a differential diagnosis. However, a generation of further evidence to support this is necessary to be able to come for a conclusion. In addition, we could apply this in other places where similar situations exist. Starting of doxycycline within the first five days of symptom is known to contribute favorable outcomes in rickettsioses [17, 20]. We believe that our high index of suspicion and starting of doxycycline without waiting to see investigation reports were key to his recovery.

This patient states that he regularly tends his cattle and dog and thus they are free of any ticks, but wild animals visit his backyard regularly. Deforestation and fragmentation of habitat combined with availability of food in the human habitat increasingly drive peridomestic greater bandicoot rats and wild animals like mongooses, porcupines, toque macaque monkeys, and feral pigs harboring the brown dog tick, the usual vector of SFG rickettsioses to the human habitat in Sri Lanka including the backyards of this patient, some of the authors, and our hospital situated in the Kandy city and that is likely to result in exchange of ticks on them with domestic animals and people [9, 10]. Recent popularity of ecotourism in the country may also contribute to this exchange. These factors and increased awareness of the infection among local clinicians resulting in the rise of diagnosed cases may be contributing to the rise of incidence of SFG rickettsioses in Sri Lanka.

## Figures and Tables

**Table 1:** Summary of his laboratory investigation results.

Investigation	Reference range										
Day of illness		Day 4	Day 5	Day 6	Day 7	Day 8	Day 9	Day 10	Day 11	Day 13	Day 14
White cell count (×10^9^/l)	4.0–10.0 × 10^9^ (l)	5.56	5.48	7.69 ⟶ 8.54	4.61 ⟶ 3.78	6.29	7.07	8.44	10.8		6.78
Neutrophils (%)	50–70%	90.5	91.8	90.4 ⟶ 88.7	84.1 ⟶ 78.9	77.1	69.9	69.9	79.4		77.2
Lymphocytes (%)	20–40%	4.5	3.9	5.3 ⟶ 4.4	9.5 ⟶ 13.8	8.0	16.5	15.9	12.4		16
Hemoglobin (g/dl)	11–16%	11.3	11.4	11.0 ⟶ 11.5	11.3 ⟶ 11.7	11.8	11.6	13.4	13.5		11.6
Hematocrit (%)	37–54%	33.5	32	32.2 ⟶ 33	32.2 ⟶ 33.1	37			40		36.1
Platelet count (×10^9^/l)	150–450 × 10^9^ (l)	30	32	7 ⟶ 12	11 ⟶ 17	37	77	120	170		257
Dengue IgM and IgG antibodies				Both were negative							
IgM against leptospires										Negative	
Serum alanine transaminase^1^ (ALT) (U/l)	7.0–28 U/l	83.9	80.6	81.8	69.5	159	39.6		36.2		31.5
Serum aspartate transaminase^2^ (AST) (U/l)	13.0–31.0 U/l	79.0	78.2	103.8	85.6	106	18.8		22.0		29.3
Serum C-reactive protein(CRP) (mg/l)	0 (*μ*mol/l)	191.5	198.6	237.0			24.8	16.1			3.9
Serum creatinine (*μ*mol/l)	59–104 (*μ*mol/l)	148.9	143.1	163.4	120.9		66				57
Blood urea (mmol/l)	2.80–7.20 (mmol/l)	12.4	12.3	14.7	15.6		7.47				
Serum sodium (mmol/l)	133–148 (mmol/l)	132					132		138		
Serum potassium (mmol/l)	3.5–5.3 (mmol/l)	4.8					3.0		3.5		
Troponin I (ng/ml)	<0.01	0.04									
Urine full report	No RBCs				80–100						
RBCs											
Blood culture			No growth after 24 hrs								
Chest X-ray		Bilateral patchy opacities									
Serum protein (g/dl)	6.4–8 (g/dl)				5.28						
Serum albumin (g/dl)	3.2–5.4 (g/dl)				3.0						
Total bilirubin (*μ*mol/l)	3–21 (*μ*mol/l)				17.9						
Bilirubin direct (*μ*mol/l)	1–7 (*μ*mol/l)				13.5						
INR					0.81						
aPTT (sec)	26–36 sec				28.6						
Throat swab for influenza A and B			Negative								
IgG against hantavirus (IFA)										Negative	
IgG against *Rickettsia conorii* (IFAT)						Positive					
IgM against *Rickettsia conorii* (IFAT)						Positive					

^1^Serum alanine transaminase (ALT) is also known as serum glutamic pyruvic transaminase (SGPT); ^2^serum aspartate transaminase (AST) is also known as serum glutamic oxaloacetic transaminase (SGOT).
